# The Role of Oxidative Stress in Common Risk Factors and Mechanisms of Cardio-Cerebrovascular Ischemia and Depression

**DOI:** 10.1155/2019/2491927

**Published:** 2019-11-15

**Authors:** Danfeng Lin, Lingling Wang, Shenqiang Yan, Qing Zhang, John H. Zhang, Anwen Shao

**Affiliations:** ^1^Department of Surgical Oncology, Second Affiliated Hospital, School of Medicine, Zhejiang University, Hangzhou, China; ^2^Department of Neurology, Second Affiliated Hospital, School of Medicine, Zhejiang University, Hangzhou, China; ^3^Department of Psychiatry, Second Affiliated Hospital, School of Medicine, Zhejiang University, Hangzhou, China; ^4^Department of Physiology and Pharmacology, Loma Linda University, Loma Linda, CA 92354, USA; ^5^Department of Neurosurgery, Second Affiliated Hospital, School of Medicine, Zhejiang University, Hangzhou, China

## Abstract

The public health sector faces a huge challenge as a result of the high prevalence and burden of disability caused by ischemic cardio-cerebrovascular disease (CVD) and depression. Although studies have explored the underlying mechanisms and potential therapies to address conditions, there is no treatment breakthrough, especially for depression which is highly influenced by social stressors. However, accumulating evidence reveals that CVD and depression are correlated and share common risk factors, particularly obesity, diabetes, and hypertension. They also share common mechanisms, including oxidative stress (OS), inflammation and immune response, cell death signaling pathway, and microbiome-gut-brain axis. This review summarizes the relationship between ischemic CVD and depression and describes the interactions among common risk factors and mechanisms for these two diseases. In addition, we propose that OS mediates the crosstalk between these diseases. We also reveal the potential of antioxidants to ameliorate OS-related injuries.

## 1. Introduction

Epidemiological data indicate that cardio-cerebrovascular diseases (CVD) and depression pose a huge global disease burden. The Global Burden of Disease 2016 (GBD 2016) study showed that CVD were the number one reason of years of life lost (YLLs) globally [1], whereas GBD 2017 demonstrated that stroke and ischemic heart disease (IHD) had the highest mortality and disability-adjusted life-years (DALYs) in China in 2017 [2]. Also, GBD 2017 reported that major depressive disorder was the third cause of years lived with disability (YLDs) after low back pain and headache disorders [3]. Therefore, there is a need to understand the underlying mechanisms and find effective therapies to control CVD and depression. A previous study demonstrated the relationship between cardio-cerebrovascular ischemia and depression [4] and clarified the vital role of oxidative stress (OS) in the pathogenesis of these two prevalent diseases. However, how OS works and its relationship with other mechanisms still remain unclear and there is little compelling evidence showing depression can cause ischemic CVD via some clear mechanisms, although many studies proved depression acting as a dependent or independent risk factor for ischemic CVD [5]. Herein, this review summarizes studies in the last five years and focuses on the risk factors and mechanisms of ischemic CVD and depression. It explores the central role of OS in the connection between the two diseases in detail, aimed at finding emerging targets in future therapies.

## 2. Overview of Cardio-Cerebrovascular Ischemia

Ischemic CVD are diseases caused by a lack of blood supply because of changes in blood vessels or blood flow, leading to multiple organ dysfunction and even death. The main types of ischemic CVD are cerebral ischemic stroke and IHD. But it is widely known that the main pathophysiology of IHD is coronary blood flow reduction caused by coronary artery atherosclerosis [6]; therefore, the term “coronary heart disease (CHD)” is often used to describe this syndrome. Thus, this review focuses on cerebral ischemia and CHD. In this section, their prevalence, common risk factors and mechanisms are discussed. Moreover, either ischemic stroke or CHD gives rise to depression.

### 2.1. Overview of Ischemic Stroke

Of all strokes, ischemic stroke accounts for 87%, and the remaining 13% are hemorrhagic strokes. According to the GBD 2016, there were about 80.1 million cerebrovascular patients worldwide, among whom 67.6 million had an ischemic stroke and 2.7 million died [4]. Generally, ischemic stroke symptoms manifest as sudden confusion or difficulty in speaking and sudden facial numbness and weakness of limbs [7, 8]. The high prevalence and disability associated with this condition are tightly associated with the risk factors and pathophysiological mechanisms, which are discussed in the next sections. As for risk factors, approximately 90% of them are health-related (such as high systolic blood pressure (BP), obesity, hyperglycemia, and hyperlipidemias), whereas 74% are behavioral risk factors and 29% are air pollution risk factors [4]. When it comes to mechanisms, they are associated with abnormal cell metabolism, cellular dysfunction, and various pathological events (such as immune responses, inflammatory reactions, apoptosis, and OS) [9, 10]. These often lead to the incomplete blood-brain barrier (BBB), loss of cell integrity, acute neuronal death, and early/secondary brain injuries [11].

Diseases of postischemic stroke including motor and sensory deficits, aphasia, and psychological distress affect patients' recovery to a large extent [12]. According to previous studies, one-third of stroke survivors experience depression, anxiety, or apathy. A systematic review and meta-analysis covering 50 studies revealed the prevalence of depression among stroke survivors to be 29% (95% confidence interval (CI) 25-32) [13]. In another meta-analysis covering 61 observational studies, the pooled frequency estimate of poststroke depression (PSD) was 31% (95% CI 28-35), though the proportional frequency varies across studies [14]. Moreover, in women, a prior history of depression and major physical disability was a significant predictor of PSD occurring within the first six months [15]. Taken together, cerebral ischemic stroke is a prevalent intractable disease with complicated mechanisms and it has a close relationship with depression, requiring effective prevention and treatment for both.

### 2.2. Overview of Cardiac Ischemia

Similar to ischemic stroke, CHD has a high incidence and some common risk factors and mechanisms at the cellular and tissue level. Data showed that CHD accounted for 43.2% of CVD deaths in 2016, including more than 3.6 million people in the United States [4]. In terms of risk factors, despite the variations in populations (e.g., age, sex, country of origin, and ethnic groups), traditional risk factors include unhealthy dietary, high systolic BP, high total cholesterol (TC) level, and high fasting plasma glucose level [16, 17]. Nevertheless, near 80% of CVD could be prevented after maintaining the levels of three risk factors mentioned above [18]. And a seventeen-year follow-up study showed that 64% female and 45% male CHD deaths could have been prevented by avoiding hypertension, smoking, and high TC (≥240 mg/dL) [19]. When it comes to mechanisms, the pathogenesis of ischemic stroke occurs in CHD patients who have extra biological defects, including cardiac autonomic dysfunction, endothelial and platelet dysfunction, and elevated catecholamine levels. These activities result in cardiac malfunctions and depression [5]. Different from the incidence of PSD, approximately 20% of CHD patients have major depression, and another 20% have minor depression at any given point in the course of their illness [5]. To sum up, CHD is a multifactor disease with complex mechanisms and its relationship with depression requires further studies.

## 3. Overview of Depression

Depression is a mental state characterized by a pessimistic sense of inadequacy and a despondent lack of activity, affecting a fairly large population worldwide. Nearly a fifth of the world's population experience one episode of depression at some point in their life, and the World Health Organization (WHO) predicted that this disease would rank the first as the cause of disease burden by 2030 [20]. Major symptoms of depression include low mood, anhedonia, poor appetite and sleep, feelings of worthless, and having suicidal thoughts. Unlike ischemic CVD, risk factors related to depression include psychosocial stressors and biological factors. The former involves factors like poor financial situation, marital status, and life events; the latter have genetic and gender predisposition and other health conditions like obesity [21] and diabetes [22]. Mechanism underlying depression includes the monoamine hypothesis, hypothalamic-pituitary-adrenal axis, inflammation, neuroplasticity, neurogenesis [20], and OS [23–26]. Recently, inflammation and neurogenesis hypotheses have gained more acceptances [27, 28].

As mentioned above, ischemic CVD leads to depression with an incidence rate of one-third in patients after stroke and one-fifth in CHD patients. In fact, depression can increase the risk for ischemic CVD; that is, depressed people are more likely to have stroke or CHD than nondepressed individuals. A 12-year follow-up study reported that depression causes a 2-fold increase in odds of stroke [29]. The results were supported by another study that showed an enhanced risk of cerebral ischemia in depressed subjects in a meta-analysis [30], and Booth et al. found that patients experiencing stressful life events had a 33% increased risk of total stroke in another meta-analysis [31]. Five meta-analyses reported a 60-80% increased risk of CHD in participants with depression [5], although there were different diagnostic questionnaires and criteria included in the studies. In summary, depression, a frequently occurring disease has a bidirectional relationship with ischemic CVD and partially shares common risk factors (such as obesity and diabetes) and mechanisms (such as inflammation and OS) with CVD, providing a new direction for future research.

## 4. Common Risk Factors among Ischemic CVD and Depression

Studies have documented that obesity, diabetes, and hypertension are common risk factors for stroke, CHD, and depression. Progression of the diseases in relation to those factors involves overactivation of OS; this is discussed in the next section.

### 4.1. Obesity

**Figure 1:**
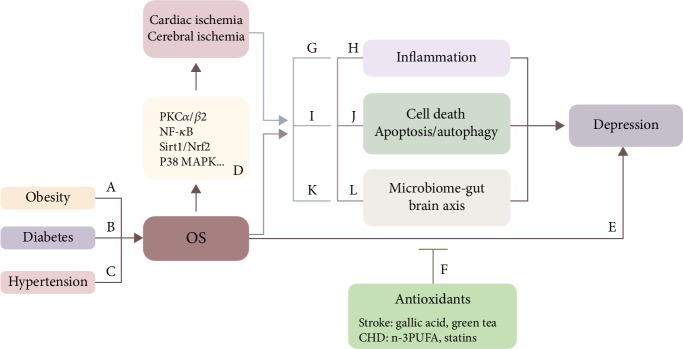
The role of OS associated with common risk factors and mechanism for ischemic CVD (stroke and CHD) and depression. A–C Obesity, diabetes, and hypertension promote the development of ischemic CVD and depression by increased OS and decreased anti-OS reactions. Moreover, this phenomenon can be reversed by using antioxidants. D OS independently affects stroke via mtDNA, oxLDL, and epithelium and affects CHD via Sirt1/Nrf2 pathway, p38 MAPK pathway, NF-*κ*B/p65 pathway, and PKC*α*/*β*2 pathway. E OS facilitates depression by NOX1-derived ROS and prooxidative and proinflammatory events. F Overactive OS contributes to depression, while administrating antioxidants ameliorates depressive symptoms by using gallic acid and green tea in stroke, as well as n-3 PUFA and statins in CHD. G Ischemic patients develop depression through inflammatory reactions. H OS can interact with inflammation through common molecules, such as GSK-3 and NLRP3 inflammasome. I A cascade of reactions in postischemic depression, when OS influences apoptosis through the Bcl-2/Bax pathway and mitochondrial dysfunction in J. K Microbiome-gut-brain axis contributes to the progression of depression in objects with ischemic CVD by impacting the immune system and brain activity as well as by spreading diseases through the enteric nervous system. L Studies have found the role of OS in promoting abnormal protein aggregation, brain lesions, and gut dysbiosis in this axis.

Obesity is defined by WHO as Body Mass Index (BMI) greater than 30 kg/m^2^. Obesity is a serious public health problem in modern society, with an increasing proportion of the obese population in developed and developing countries by years. Studies have confirmed that obesity is a complicated disease that increases the risk of chronic diseases such as CVD, diabetes, and depression. In particular, experiments have demonstrated that obesity as a state of chronic inflammation with amplified OS plays a role in the occurrence of ischemic stroke [32, 33], and a nationwide population-based study concluded that obesity, especially the metabolically unhealthy type, raised the risk of stroke [34]. Besides ischemic stroke, obesity is considered as an independent risk factor for cardiovascular disease. However, recent epidemiological data have revealed a growing interest in “obesity paradox” theory; that is, overweight and mildly obese individuals may have a decreased or similar outcome of mortality compared with their normal-weight counterparts after CVD has been established, despite the increased risk of developing CVD in the obese. This theory stresses the importance of classifying obesity when talking about obesity-related diseases [35, 36]. Similar to the effect of obesity on CVD, there was a significant association between obesity and depression and more severe depressive symptoms were observed in the obese group compared with a normal-weight group, based on a meta-analysis of 18 studies that enrolled 51,272 participants [37]. Therefore, obesity is associated with ischemic CVD and depression. After investigating the mechanisms of obesity in these disorders, a vast array of data from human studies have indicated that there are complex pathological processes influenced by environmental, genetic, and epigenetic interactions [38]. Mechanisms like inflammation and gut-brain axis which are involved in the pathogenesis and OS (Figure 1 A) also play a crucial role [39].

### 4.2. Diabetes

Diabetes is a variable carbohydrate metabolic disorder caused by a combination of genetic and environmental factors, usually characterized by insufficient secretion or utilization of insulin. The International Diabetes Federation (IDF) recently reported that about 425 million adults worldwide suffered from diabetes in 2017. China has the largest number of diabetic patients (114.4 million) in the world. As a serious chronic disease, diabetes causes many complications and increases the risks of acquiring other diseases, including ischemic CVD and depression, if not managed. Diabetes contributes to ischemic CVD. After correction for other risk factors, diabetics have more than double the risk of ischemic stroke compared to nondiabetics, and stroke accounts for approximately 20% of deaths in diabetics [40–42]. Also, prediabetics and the duration of diabetes increase stroke risk [7]. Dysregulation of metabolism in diabetes adversely affects vascular wall, and this causes vascular dysfunction, making patients more prone to atherosclerosis; this significantly increases their risk of myocardial infarction (MI) and CHD [43]. The findings are supported by an observation of a cohort CVD study with 71,745.4 person-years, showing that diabetes had a significant impact on the residual lifetime risk of CHD in middle-aged men and women [44]. Diabetes promotes the onset and progression of depression. People with diabetes (either type 1 or type 2) are twice likely to have depression compared to nondiabetics [45]. This is explained by two possible mechanisms. The first one is the psychosocial burden of chronic disease [46], whereas the second one is connected to biochemical changes in diabetes, such as inflammatory and psychoneuroendocrine mechanisms [47, 48]. Presently, diabetes affects a large population worldwide and has connections with both CVD and depression.

The underlying mechanisms explaining this relationship are similar to those of obesity. The brain is susceptible to glucose, and the risk of stroke increases by OS through complex mechanisms [49, 50], and diabetes worsens results of acute coronary syndrome due to overactivation of OS [43]. Recent studies have proved that reducing OS response via antioxidant treatment is able to ease the depressive-like behavior caused by diabetes [51] and implicated gut microbiota as a common mechanism mediated by OS in diabetes and depression [23, 52]. In conclusion, diabetes is an important risk factor for ischemic CVD and depression, and its pathogenesis is closely related to OS (Figure 1 B).

### 4.3. Hypertension

Two recent studies published in The Lancet on hypertension control in high-, middle-, and low-income countries revealed an increasing trend of hypertension prevalence over time [53]. It is widely acknowledged that high BP is one of the most important risk factors for stroke and heart disease [54]. Several studies have demonstrated a significant increase in the risk of depression in hypertension patients. In cases of stroke, there is a strong, direct, linear, and continuous relationship between high BP and stroke risk [7]. The mechanism of ischemic stroke caused by hypertension has been relatively clear, among which OS (Figure 1 C) occupies an important position [55]. When considering heart disease, detailed studies over the past few decades have identified hypertension as a leading cause of CHD, especially myocardial ischemia. Importantly, the formation of atherosclerotic plaques, associated with OS, was involved in many processes, from the classic chain reaction of hypertension to the development of CHD and myocardial ischemia [56]. Parallel to CVD, depression has links with high BP. A Dutch study showed that depressed elderly patients with higher BP showed more symptoms of apathy than the controls with normal BP [57]. A Latin American study illustrated that even after adjusting for many potential confounders, hypertension was an independent predictor of depressive symptoms among Mexican community-dwelling elders [58]. In short, hypertension is another important common risk factor for the mentioned illnesses and OS also takes part in the pathological processes.

## 5. The Inherent Connection between Risk Factors and Mechanisms of Ischemic CVD and Depression

This section provides a brief overview of the common mechanisms associated with ischemic CVD and depression, describes the inherent connection among risk factors, pathological changes, and the two disorders, and identifies the central role of OS in the network (Figure 1). In recent years, these mechanisms mentioned earlier including OS, inflammation and immune response, cell death signaling pathway, and microbiome-gut-brain axis can be mediated by OS.

### 5.1. Oxidative Stress and Antioxidative System

Oxidation-reduction is beneficial for physiological functions under normal conditions, and imbalanced redox resulting from excessive productions of free radicals (FR) and weakened antioxidative defenses can cause multiple pathological pathways, inducing mitochondrial dysfunction and destruction of homeostasis. Excess production of reactive oxygen species (ROS), especially mitochondrial ROS (mitoROS) produced by mitochondrial respiration, is detrimental to body health. Redundant mitoROS exert an adverse effect on metabolic pathways, such as molecular oxidation and impairment of ATP synthesis [59]. Subsequently, there is intracellular ionic imbalance and activation of intracellular proteases, lipases, and ribonucleases due to energy deficits, which is fatal to cells and organs. Moreover, overactive Nicotinamide Adenine Dinucleotide Phosphate (NADPH) oxidase (NOX) can bring an excess of superoxide [60] and hydroxyl radicals and ions to aggravate injuries [61]. In contrast, reduced antioxidant levels are negatively correlated with prognosis in some diseases like CVD and depression. Since nonenzymatic substances (e.g., melatonin and ursolic acid) can detoxify ROS [62], and antioxidant enzymes (e.g., superoxide dismutase) and catalase (CAT) have effects on scavenging superoxide radicals; declined levels of those antioxidants negatively affect health in varying degrees [63, 64]. Inhibition or lack of nuclear factor erythroid 2-related factor (Nrf2) which recognizes the antioxidant response element (ARE) and protects cells from ROS accumulation may increase injury to patients; however, this can be reversed by administration of antioxidants [65], such as dimethyl fumarate and monomethyl fumarate [66]. It is therefore not surprising that alterations in OS and anti-OS levels are found in ischemic CVD and depression as discussed earlier.

Oxidative stress can independently and directly affect stroke, CHD (Figure 1 D) and depression (Figure 1 E). After cerebral ischemia induction, energy expenditure in the brain can promote OS activity resulting in DNA damage and molecular peroxidation. For example, a case-control study about cerebral ischemia presented a significant association between elevated ROS and low mitochondrial DNA (mtDNA) in peripheral blood leukocytes [67]. Another biomarker is oxidized low-density lipoprotein (oxLDL) whose increase revealed a worse prognosis [68] and a higher prevalence of cognitive impairment in stroke survivors than the controlled group [69]. Moreover, ROS impede blood circulation and destroy the epithelium by affecting the blood vessels, hence triggering their overreaction to contractile agents, with observations of increased platelet aggregation, endothelial cell permeability, and focal endothelial cell lesions [70, 71]. In CHD, OS may contribute to vascular epithelial injury via the Sirt1/Nrf2 and p38 MAPK pathways [72], as well as the NF-*κ*B/p65 pathway [73]. Moreover, NOX activation facilitates the initiation and progression of coronary artery disease (CAD) through the PKC*α*/*β*2 signaling pathway [74]. Similar mechanisms involving active OS action also happens in depression. Studies indicate that NOX1-derived ROS induces the oxidation of NMDA receptor 1 (NR1) in the prefrontal cortex to facilitate depressive-like behaviors [75], whereas a longitudinal study suggests a cascade of prooxidative and proinflammatory events in the development of depression [76].

Furthermore, OS acts as a link between ischemic CVD and depression **(**Figure 1 F). There is a positive correlation between an elevated serum malondialdehyde (MDA) level at admission and an increased risk of depression after acute stroke, especially minor stroke [77]. Nevertheless, antioxidants can attenuate this correlation. Gallic acid [78], *Hypericum androsaemum* L. [79], and green tea induce a reduction in depressive symptoms and OS, restoring normal behavior and antioxidant endogenous defenses [80]. Similarly, greater depressive symptoms related to higher OS can be treated using omega-3 polyunsaturated fatty acid (n-3 PUFA); this mitigates OS and thus improves depression in CAD patients [81]. Also, the use of statins reduces the risk of depression in individuals after a heart attack, supporting the role of oxidative and inflammatory processes in depression [82].

To sum up, OS directly influences the pathogenesis of ischemic CVD or depression through various signaling pathways, and it also acts as a link between ischemic CVD and depression. Of note, anti-OS may open the door to rational and novel therapies for the two diseases.

### 5.2. Oxidative Stress and Risk Factors

As mentioned above, OS has relations with the three risk factors of CVD and depression and this section provides an in-depth discussion.

#### 5.2.1. Obesity

Animal and human studies have identified the relationship between obesity and OS **(**Figure 1 A). Cerebral ischemia in gerbil study revealed that there were elevated levels of OS indicators (dihydroethidium and 8-hydroxyguanine (8-OHdG)) and reduced levels of antioxidant enzymes (superoxide dismutase (SOD1) and SOD2) in the obese gerbils compared to nonobese, both in pre- and postischemic phases. But this obesity-induced oxidative damage could be attenuated by pretreated fucoidan which had antioxidant properties [83]. In CHD, research suggests that obese patients with myocardium are more susceptible to ischemia compared to nonobese people, with enhanced levels of ROS and ROS-producing enzymes (i.e., p47phox, xanthine oxidase) and reduced antioxidant activity (mitochondrial aldehyde dehydrogenase and heme oxygenase-1) [84]. In depression, obese mice fed with high-fat diet had severe depressive behaviors, which could be reversed by ondansetron treatment via restoration of brain prooxidant/antioxidant balance [85] and by allicin via activation of the Nrf2 pathway [86]. Apart from preclinical investigations, human studies have indicated that postpartum depression affects one in seven women, and obese women have an increased risk of depression through neurooxidation and neuronitrosation [87, 88].

#### 5.2.2. Diabetes

Diabetes can affect ischemic CVD and depression via OS, and antioxidant therapy possibly improves these conditions (Figure 1 B). Whereas a study emphasized the protection of glucagon-like peptide 1 (GLP-1) agonists against oxidative and apoptotic damage in a diabetic mouse model [89], another study supported the role of metformin in improving neurological functions and OS status via the AMPK/mTOR signaling pathway in acute stroke patients with type 2 diabetes [90]. In heart disease, a clinical trial enrolling 3766 adults with prevalent diabetes mellitus illustrated that increased levels of 8-oxo-2′-deoxyguanosine, a biomarker of oxidative DNA damage, were independently associated with elevated cardiovascular mortality [91]. In depression, recent studies have proposed that one possible mechanism linking diabetes and depression was the increase in lipid peroxidation and decrease in antioxidant activity in the hippocampal and prefrontal cortices, which are the areas of the brain associated with mood [92, 93].

#### 5.2.3. Hypertension

Altered levels of antioxidants and oxidative biomarkers have demonstrated the role of OS in ischemic or depressed patients with hypertension (Figure 1 C). In salt-loaded stroke-prone spontaneously hypertensive rat (SHRSP), OS from multiple sources affected its stroke susceptibility [94]. However, curcumin could delay the occurrence of stroke and improve the survival of SHRSP through decreasing ROS levels as well as improving endothelium-dependent relaxation of the carotid artery via uncoupling the protein 2 signaling pathway [95, 96]. In heart disease, studies found that pomegranate peel extract may alleviate CHD caused by hypertension by reducing coronary angiotensin-converting enzyme (ACE), OS, and vascular remodeling [97], which also occurs in linagliptin [98]. In patients with depression and hypertension, there are decreased glutathione peroxidase-1 (GPx-1) and SOD-1 activities but increased concentrations of MDA and H_2_O_2_, in comparison with the controls [99]. These results have revealed the role of OS in risk factors for CVD and depression to a full extent.

### 5.3. Oxidative Stress and Other Mechanisms

Many studies have shown the involvement of several mechanisms in CVD and depression, including inflammatory and immune response, apoptosis and autophagy, and microbiome-gut-brain axis. Although all can affect disease independently, they may act as a link between the two diseases and may interact with OS or other signaling pathways to aggravate the diseases.

#### 5.3.1. Inflammation and Immune Response

Inflammatory processes and immunoreaction participate in the pathogenesis of ischemic stroke [100], CHD [101], and depression, and they have common molecules with OS. Inflammation and immune reactions exist in ischemic CVD and depression. Initiated by stagnant blood flow after ischemic stroke, activation of intravascular leukocytes and the release of proinflammatory mediators trigger inflammation which decreases the integrity of the BBB leading to the release of danger-/damage-associated molecular patterns (DAMP) from injured neurons. This in turn induces the production of cytokines like interleukin- (IL-) 1*β* and tumor necrosis factor (TNF). Such a process then feeds back into the inflammatory cascade via cytokine and chemokine, thereby causing great damages [102]. In ischemic stroke survivors, Ferrarese et al. observed peak levels of TNF-*α* at day 1 and IL-6 and TNF-*α* at day 4, as well as a long-lasting activation of these two cytokines in peripheral blood cells [103]. Innate and adaptive immune responses also occur in ischemia, accompanied by the activation of microglia [104], neutrophils, T cells, and B cells [105]. Elsewhere, multiple human translational and preclinical studies have revealed that inflammation facilitates the development of atherosclerosis and evokes immunoreactions via the release of cytokines, such as TNF-*α*, IFN-*γ*, and IL-6 [106]. Similar to ischemic CVD, numerous studies have suggested that inflammatory and immune processes occur in depression. For example, higher IL-6, IL-18, and C-reactive protein (CRP) levels have been found in depressed individuals [107, 108]. Moreover, a study indicated that assembly of the inflammasome can act as a key point between the inflammation and immune activities [109], and depression was shown to be in connection with proinflammatory activation of the peripheral innate immune system with relative inactivation of the adaptive immune system [110].

Inflammation and immune response may be a link between ischemic CVD and depression (Figure 1 G). These mechanisms facilitate the progression of depression in patients after ischemic CVD. For instance, a 2-year prospective study showed that depression was prevalent in patients after ischemic stroke, and IL-6 was positively correlated with the risk of PSD [111]. As for CHD subjects, results conveyed elevated levels of inflammation, manifested as higher levels of CRP, IL-6, and plasma vascular endothelial cell growth factor (VEGF), in CHD patients with depression under the condition of hypothalamic-pituitary-adrenocortical (HPA) axis hypoactivity and activation of the kynurenine pathway [112]. On the contrary, animals receiving Resolvin D1 which is a metabolite of n-3 PUFA that can diminish neutrophil accumulation in the ischemic myocardium showed decreased depression-like symptoms via better performance in the forced swim tests [113]. This mechanism increases the risk of ischemic CVD among depressed individuals [5].

Oxidative stress can mediate inflammatory and immune activities in the mentioned diseases (Figure 1 H). In cerebral ischemia, ROS leads to the phosphorylation of glycogen synthase kinase 3 (GSK-3) which inhibits nuclear translocation of element-binding protein (CREB) and Nrf2-ARE pathway, leading to elevated levels of proinflammatory and inflammatory cytokines [114, 115]. Besides, oxidative damage to platelets and endothelial cells is also involved in the inflammatory response, with overactive leucocyte activities [116]. Similarly, in depressed individuals, OS took part in inflammatory reactions by activating NOD-like receptor protein 3 (NLRP3) inflammasome to activate IL-1*β* and IL-18 [117].

#### 5.3.2. Cell Death Signaling Pathway

What is generally known is that the cell death signaling pathway is closely associated with the development of ischemic CVD and depression, and it relates to OS. Firstly, cell death, especially apoptosis and autophagy, is frequently observed in the two disorders. The receptor-interacting protein 1 kinase- (RIP1K-) mediated necroptosis contributes to neuronal and astrocytic cell death in ischemic stroke via the autophagic-lysosomal pathway [118], and the p53-dependent pathway and the calpain-caspase-3 pathway play a part in apoptosis of neural cells [119, 120]. In comparison, endothelial progenitor cells facilitate coronary atherosclerotic heart disease via autophagy and activation of the mTOR signaling pathway [121]. However, myocardial cathepsin D can protect against cardiac remodeling and malfunction through promoting myocardial autophagic flux [122], and Phellinus Linteus Mycelium (PLM) alleviates myocardial ischemia-reperfusion by suppressing proapoptotic signaling and regulation of autophagic signaling [123]. Similar to ischemic CVD, cell death signaling has been observed in depression. The TNF-like weak inducer of apoptosis (TWEAK) induces apoptosis in resident brain cells in the cortex and hippocampus to cause lupus-associated neurobehavioral deficits including depression and cognitive dysfunction [124], whereas chronic stress induces depressive-like behavior and hippocampal neuropathology by regulating autophagy via the PI3K/Akt/mTOR signaling [125].

Secondly, cell death may link ischemic CVD and depression (Figure 1 I), although evidence for this comes from few studies. In a bilateral internal carotid artery occlusion mouse model, cerebral ischemia causes depressive-like behaviors through the caspase-8/9-dependent neural cell apoptosis [126]. In patients with acute myocardial infarction comorbid with depression, escitalopram may directly confer cardio-protection by inhibiting proapoptotic pathways [127].

Thirdly, OS mediates the cell death signaling pathway, especially apoptosis, to affect the mentioned diseases (Figure 1 J). Several studies have shown that ROS could stimulate apoptosis, necrosis, and their combined pathway in ischemia [128], possibly by mediating antiapoptotic protein, B cell lymphoma-2 (Bcl-2), and the proapoptotic apoptosis regulator, Bcl-2-associated X protein (Bax) protein [129]. Furthermore, OS mainly correlates with intrinsic apoptosis. After OS-induced mitochondrial dysfunction, the released cytochrome c (Cytc) binds to the apoptotic protease to form apoptosomes, contributing to DNA damage and apoptotic cell death [129]. Similar to mechanisms in ischemia, caspase-9 is activated and apoptosome is formed to induce apoptosis in depressed animals [130]. In terms of autophagy, it has been reported that excessive ROS adjusts the transcriptional regulatory mechanisms in the nucleus to promote autophagy, but the underlying mechanisms are not well known [131].

#### 5.3.3. Microbiome-Gut-Brain Axis

Recent studies have focused on the microbiome-gut-brain axis. The microbiome-gut-brain axis plays a role in either ischemic CVD or depression. It has been reported that after stroke, intestinal dysbiosis not only alters the immune balance of the small intestine with an increase in regulatory T cells and a reduction in interleukin- (IL-) 17-positive *γδ* T cells but also suppresses trafficking of effector T cells from the gut to the leptomeninges [132, 133]. In the myocardial infarction model after three-week arterial occlusion, rats began to exhibit depression-like symptoms with increased gastric retention rates [134]. In depression, after surveying a large microbiome population cohort and analyzing fecal metagenomes, researchers indicated a potential role of microbial *γ*-aminobutyric acid production and provided population-scale pieces of evidence for microbiome relating to mental health [135].

The latest studies have documented that the microbiome-gut-brain axis can be a link between ischemic CVD and depression (Figure 1 K). The 2017 named series and the psychoneuroimmunology research reported that commensal microbes may impact the immune system and brain activity via behavioral and immunological responses to social stresses, which can be attenuated by healthy dietary [136]. Moreover, the enteric nervous system (ENS) structure and neurochemistry are similar to the CNS; therefore, pathogenic mechanisms inducing CNS disorders might also give rise to ENS dysfunction and nerves, interconnecting the ENS and CNS, can be conduits for spreading of disease [137]. This collection of work describes a connection between the microbiota, brain, behavior, immunity, stroke, and depression, to provide an important rationale for extending the work in the future. Contrarily, a study showed that depressive disorder and gastrointestinal dysfunction after myocardial infarction were associated with abnormal metabolism of tryptophan-5-hydroxytryptamine, an important substance in the gut-brain axis [134].

Oxidative stress also plays a role in regulating the microbiome-gut-brain axis related to ischemia and depression (Figure 1 L). Intestinal microbiome might increase ROS and promote abnormal protein aggregation and brain lesions, which can result in alteration of gut properties and microbiota [138]. For instance, ischemic stroke intensifies gut barrier breakdown by overproduced ROS and aggravates microbiota alterations, followed by the translocation of a selective bacterial strain to the surrounding tissues. As a consequence, it promotes poststroke infections [139]. In depression, several studies suggest that ROS has connections with gut dysbiosis [140], one of which illustrates that increased chronic apical periodontitis and lipopolysaccharide levels probably lead to depression via OS-induced hypernitrosylation and neuroprogressive processes [141].

## 6. Conclusion

**Figure 2:**
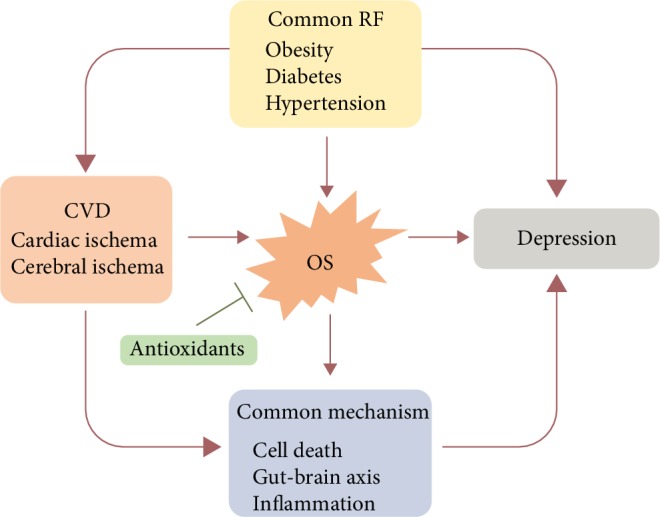
The relationship among OS, common risk factors, and common mechanism of ischemic CVD (stroke and CHD) and depression. The common risk factors, obesity, diabetes, and hypertension, can affect either ischemic stroke or depression. Moreover, they can exert influence via overactive OS activity and downregulated antioxidant defenses. On the other hand, OS acts as one of the common mechanisms promoting postischemic depression and it also interacts with inflammation, cell death signaling pathway (apoptosis and autophagy), and gut-brain axis to exacerbate the process of postischemic depression.

Based on the mechanisms described in the previous sections, we make the following conclusions (Figure 2). Notably, ischemic CVD and depression are highly prevalent and are major causes of disability. These two disorders are interrelated; that is, stroke survivors are likely to develop depression and depressed individuals have a higher risk for stroke and CHD than nondepressed people [29–31]. Additionally, these two diseases share some common risk factors, such as obesity, diabetes, and hypertension. The underlying mechanisms involved in many pathological processes, such as inflammation, cell death (apoptosis and autophagy), microbiome-gut-brain axis, and OS, also participate in both diseases. Notably, OS is in the center of this web. These three risk factors are associated with OS and can trigger the development and aggravate the progression of these diseases via overactivation of OS and attenuation of antioxidant activities. In addition, OS directly increases the risk of depression in patients with cardiovascular diseases, whereas it increases the risk of cardiovascular diseases in depressed people. Thirdly, high levels of ROS promote the pathogenesis of ischemic CVD and depression via OS-induced inflammation, cell death signaling, and microbiome-gut-brain axis. In summary, the common risk factors increase the production of OS and reduce antioxidant defenses, thereby promoting the occurrence and development of interacted ischemic CVD and depression. Application of antioxidants can mitigate OS-related injuries and diseases. Due to the central role of OS in these two illnesses, inhibition of OS may provide novel and promising therapeutic strategies for the two conditions.

## 7. Limitations and Perspectives

The previous studies investigating the relationship between hypertension and depression have been limited to the elderly population, and some meta-analyses have contradictory results due to high heterogeneity and other reasons such as the following: (1) The clinical manifestation of some types of stroke or depression varies among patients, and there may be some biases when studying such patients. (2) The small sample size or short follow-up time of some studies may introduce some variations in the results. This requires further longitudinal, large-sample size, cohort studies to provide more conclusive outcomes.

The inconsistency among clinical diagnostic criteria and the lack of uniform and timely evaluation tools may increase errors in the data, which makes head-to-head comparisons among studies unsuitable. In particular, there are many types of depression which are diagnosed with different diagnostic guidelines. This will reduce the comparability among studies.

Many animal experiments cannot adequately eliminate the interference of other factors, and hence, the impact of certain factors on disease phenotypes may affect the results [5]. This calls for the development of precise and stable animal models of stroke or depression, which will provide accurate assessment of disease progression and pathogenesis.

Despite the mechanisms discussed in this review, there are no clear cellular or molecular level pathways that explain the pathogenesis of stroke and depression. Therefore, appropriate in vitro models of apoplexy or depression are urgently required.

Some antioxidants exert anti-inflammatory, antiapoptotic, and antioxidative effects simultaneously. So when they have certain curative effects on diseases, they cannot be fully explained only by antioxidation effect [79–82]. This also suggests further studies are required to reveal other mechanisms of oxidative stress in diseases.

According to the data reviewed in this article, we propose the following research directions: (1) Should antioxidation therapy be given to depression patients to prevent ischemic cardiovascular disease? Are patients with ischemic stroke and myocardial infarction suitable for antioxidant therapy to prevent later depression? (2) What is the timing of antioxidant intervention? (3) Will a combination of antioxidant therapy produce better outcomes in patients with depression or cardiovascular and cerebrovascular diseases? All of these questions are yet to be answered, and therefore, future multicenter randomized controlled clinical studies with large samples and more scientific and reasonable experimental verification are needed.

## References

[1] Moraga P., GBD 2016 Causes of Death Collaborators (2017). Global, regional, and national age-sex specific mortality for 264 causes of death, 1980-2016: a systematic analysis for the Global Burden of Disease Study 2016. *The Lancet*.

[2] Zhou M., Wang H., Zeng X. (2019). Mortality, morbidity, and risk factors in China and its provinces, 1990-2017: a systematic analysis for the Global Burden of Disease study 2017. *Lancet*.

[3] James S. L., Abate D., Abate K. H. (2018). Global, regional, and national incidence, prevalence, and years lived with disability for 354 diseases and injuries for 195 countries and territories, 1990-2017: a systematic analysis for the Global Burden of Disease Study 2017. *Lancet*.

[4] Benjamin E. J., Muntner P., Alonso A. (2019). Heart disease and stroke statistics-2019 update: a report from the American Heart Association. *Circulation*.

[5] Carney R. M., Freedland K. E. (2017). Depression and coronary heart disease. *Nature Reviews Cardiology*.

[6] Mieres J. H., Bonow R. O. (2016). Ischemic heart disease in women: a need for sex-specific diagnostic algorithms. *JACC: Cardiovascular Imaging*.

[7] Boehme A. K., Esenwa C., Elkind M. S. (2017). Stroke risk factors, genetics, and prevention. *Circulation Research*.

[8] Dong S., Maniar S., Manole M. D., Sun D. (2018). Cerebral hypoperfusion and other shared brain pathologies in ischemic stroke and Alzheimer’s disease. *Translational Stroke Research*.

[9] Yu I. C., Kuo P. C., Yen J. H. (2017). A combination of three repurposed drugs administered at reperfusion as a promising therapy for postischemic brain injury. *Translational Stroke Research*.

[10] Yigitkanli K., Zheng Y., Pekcec A., Lo E. H., van Leyen K. (2017). Increased 12/15-lipoxygenase leads to widespread brain injury following global cerebral ischemia. *Translational Stroke Research*.

[11] Jiang X., Andjelkovic A. V., Zhu L. (2018). Blood-brain barrier dysfunction and recovery after ischemic stroke. *Progress in Neurobiology*.

[12] Hackett M. L., Köhler S., O’Brien J. T., Mead G. E. (2014). Neuropsychiatric outcomes of stroke. *Lancet Neurology*.

[13] Ayerbe L., Ayis S., Wolfe C. D., Rudd A. G. (2013). Natural history, predictors and outcomes of depression after stroke: systematic review and meta-analysis. *The British Journal of Psychiatry*.

[14] Hackett M. L., Pickles K. (2014). Part I: frequency of depression after stroke: an updated systematic review and meta-analysis of observational studies. *International Journal of Stroke*.

[15] Guiraud V., Gallarda T., Calvet D. (2016). Depression predictors within six months of ischemic stroke: the DEPRESS study. *International Journal of Stroke*.

[16] Global Burden of Cardiovascular Diseases Collaboration, Roth G. A., Johnson C. O. (2018). The burden of cardiovascular diseases among US states, 1990-2016. *JAMA Cardiology*.

[17] Hippisley-Cox J., Coupland C., Brindle P. (2017). Development and validation of QRISK3 risk prediction algorithms to estimate future risk of cardiovascular disease: prospective cohort study. *BMJ*.

[18] Yang Q., Cogswell M. E., Flanders W. D. (2012). Trends in cardiovascular health metrics and associations with all-cause and CVD mortality among US adults. *JAMA*.

[19] Mensah G. A., Brown D. W., Croft J. B., Greenlund K. J. (2005). Major coronary risk factors and death from coronary heart disease: baseline and follow-up mortality data from the Second National Health and Nutrition Examination Survey (NHANES II). *American Journal of Preventive Medicine*.

[20] Malhi G. S., Mann J. J. (2018). Depression. *Lancet*.

[21] Milaneschi Y., Lamers F., Peyrot W. J. (2017). Genetic association of major depression with atypical features and obesity-related immunometabolic dysregulations. *JAMA Psychiatry*.

[22] Chen S., Zhang Q., Dai G. (2016). Association of depression with pre-diabetes, undiagnosed diabetes, and previously diagnosed diabetes: a meta-analysis. *Endocrine*.

[23] Luca M., di Mauro M., di Mauro M., Luca A. (2019). Gut Microbiota in Alzheimer's Disease, Depression, and Type 2 Diabetes Mellitus: The Role of Oxidative Stress. *Oxidative Medicine and Cellular Longevity*.

[24] Jiang C. Y., Qin X. Y., Yuan M. M., Lu G. J., Cheng Y. (2018). 2,3,5,4-Tetrahydroxystilbene-2-O-beta-D-glucoside Reverses Stress-Induced Depression via Inflammatory and Oxidative Stress Pathways. *Oxidative Medicine and Cellular Longevity*.

[25] Vaváková M., Ďuračková Z., Trebatická J. (2015). Markers of oxidative stress and neuroprogression in depression disorder. *Oxidative Medicine and Cellular Longevity*.

[26] Wu Y., Wang L., Hu K. (2018). Mechanisms and therapeutic targets of depression after intracerebral hemorrhage. *Frontiers in Psychiatry*.

[27] Setiawan E., Wilson A. A., Mizrahi R. (2015). Role of translocator protein density, a marker of neuroinflammation, in the brain during major depressive episodes. *JAMA Psychiatry*.

[28] Molendijk M. L., Spinhoven P., Polak M., Bus B. A., Penninx B. W., Elzinga B. M. (2014). Serum BDNF concentrations as peripheral manifestations of depression: evidence from a systematic review and meta-analyses on 179 associations (*N*=9484). *Molecular Psychiatry*.

[29] Jackson C. A., Mishra G. D. (2013). Depression and risk of stroke in midaged Women. *Stroke*.

[30] Pan A., Sun Q., Okereke O. I., Rexrode K. M., Hu F. B. (2011). Depression and risk of stroke morbidity and mortality: a meta-analysis and systematic review. *JAMA*.

[31] Booth J., Connelly L., Lawrence M. (2015). Evidence of perceived psychosocial stress as a risk factor for stroke in adults: a meta-analysis. *BMC Neurology*.

[32] Chen S. D., Yang D. I., Lin T. K., Shaw F. Z., Liou C. W., Chuang Y. C. (2011). Roles of oxidative stress, apoptosis, PGC-1*α* and mitochondrial biogenesis in cerebral ischemia. *International Journal of Molecular Sciences*.

[33] Radak D., Resanovic I., Isenovic E. R. (2014). Link between oxidative stress and acute brain ischemia. *Angiology*.

[34] Lee H. J., Choi E. K., Lee S. H., Kim Y. J., Han K. D., Oh S. (2018). Risk of ischemic stroke in metabolically healthy obesity: a nationwide population-based study. *PLoS One*.

[35] Jahangir E., De Schutter A., Lavie C. J. (2014). The relationship between obesity and coronary artery disease. *Translational Research*.

[36] Lavie C. J., Milani R. V., Ventura H. O. (2009). Obesity and cardiovascular disease: risk factor, paradox, and impact of weight loss. *Journal of the American College of Cardiology*.

[37] Quek Y. H., Tam W. W. S., Zhang M. W. B., Ho R. C. M. (2017). Exploring the association between childhood and adolescent obesity and depression: a meta‐analysis. *Obesity Reviews*.

[38] Haslam D. W., James W. P. (2005). Obesity. *Lancet*.

[39] Santilli F., Guagnano M. T., Vazzana N., la Barba S., Davi G. (2015). Oxidative stress drivers and modulators in obesity and cardiovascular disease: from biomarkers to therapeutic approach. *Current Medicinal Chemistry*.

[40] Banerjee C., Moon Y. P., Paik M. C. (2012). Duration of diabetes and risk of ischemic stroke the Northern Manhattan Study. *Stroke*.

[41] Luitse M. J. A., Biessels G. J., Rutten G. E. H. M., Kappelle L. J. (2012). Diabetes, hyperglycaemia, and acute ischaemic stroke. *Lancet Neurology*.

[42] Sui X., Lavie C. J., Hooker S. P. (2011). A prospective study of fasting plasma glucose and risk of stroke in asymptomatic men. *Mayo Clinic Proceedings*.

[43] Beckman J. A., Creager M. A., Libby P. (2002). Diabetes and atherosclerosis - epidemiology, pathophysiology, and management. *Jama, the journal of the American Medical Association*.

[44] Turin T. C., Okamura T., Rumana N. (2017). Diabetes and lifetime risk of coronary heart disease. *Primary Care Diabetes*.

[45] de Groot M., Anderson R., Freedland K. E., Clouse R. E., Lustman P. J. (2001). Association of depression and diabetes complications: a meta-analysis. *Psychosomatic Medicine*.

[46] Knol M. J., Heerdink E. R., Egberts A. C. (2007). Depressive symptoms in subjects with diagnosed and undiagnosed type 2 diabetes. *Psychosomatic Medicine*.

[47] Herder C., Fürstos J. F., Nowotny B. (2017). Associations between inflammation-related biomarkers and depressive symptoms in individuals with recently diagnosed type 1 and type 2 diabetes. *Brain Behavior and Immunity*.

[48] Stuart M. J., Baune B. T. (2012). Depression and type 2 diabetes: inflammatory mechanisms of a psychoneuroendocrine co-morbidity. *Neuroscience and Biobehavioral Reviews*.

[49] Aldini G., Dalle-Donne I., Facino R. M., Milzani A., Carini M. (2007). Intervention strategies to inhibit protein carbonylation by lipoxidation‐derived reactive carbonyls. *Medicinal Research Reviews*.

[50] Cobley J. N., Fiorello M. L., Bailey D. M. (2018). 13 reasons why the brain is susceptible to oxidative stress. *Redox Biology*.

[51] Réus G. Z., dos Santos M. A. B., Abelaira H. M. (2016). Antioxidant treatment ameliorates experimental diabetes‐induced depressive‐like behaviour and reduces oxidative stress in brain and pancreas. *Diabetes-Metabolism Research and Reviews*.

[52] Moulton C. D., Pickup J. C., Ismail K. (2015). The link between depression and diabetes: the search for shared mechanisms. *The Lancet Diabetes & Endocrinology*.

[53] Zhou B., Danaei G., Stevens G. A. (2019). Long-term and recent trends in hypertension awareness, treatment, and control in 12 high-income countries: an analysis of 123 nationally representative surveys. *Lancet*.

[54] Lu Y., Ezzati M., Danaei G., Kclose H. (2012). Global burden of metabolic risk factor of chronic diseases collaborating group (mediated effect of adiposity). *American Journal of Epidemiology*.

[55] Sierra C., Coca A., Schiffrin E. L. (2011). Vascular mechanisms in the pathogenesis of stroke. *Current Hypertension Reports*.

[56] Vassalle C., Petrozzi L., Botto N., Andreassi M. G., Zucchelli G. C. (2004). Oxidative stress and its association with coronary artery disease and different atherogenic risk factors. *Journal of Internal Medicine*.

[57] Moonen J. E. F., de Craen A. J. M., Comijs H. C., Naarding P., de Ruijter W., van der Mast R. C. (2015). In depressed older persons higher blood pressure is associated with symptoms of apathy. The NESDO study. *International Psychogeriatrics*.

[58] García-Fabela L., Melano-Carranza E., Aguilar-Navarro S., García-Lara J. M. A., Gutiérrez-Robledo L. M., Ávila-Funes J. A. (2009). Hypertension as a risk factor for developing depressive symptoms among community-dwelling elders. *Revista De Investigacion Clinica-Clinical and Translational Investigation*.

[59] Ago T., Kuroda J., Pain J., Fu C., Li H., Sadoshima J. (2010). Upregulation of Nox4 by hypertrophic stimuli promotes apoptosis and mitochondrial dysfunction in cardiac myocytes. *Circulation Research*.

[60] Bedard K., Krause K. H. (2007). The NOX family of ROS-generating NADPH oxidases: physiology and pathophysiology. *Physiological Reviews*.

[61] Serrander L., Cartier L., Bedard K. (2007). NOX4 activity is determined by mRNA levels and reveals a unique pattern of ROS generation. *Biochemical Journal*.

[62] Reiter R. J., Tan D. X., Galano A. (2014). Melatonin: exceeding expectations. *Physiology (Bethesda)*.

[63] Wigner P., Czarny P., Synowiec E. (2018). Variation of genes involved in oxidative and nitrosative stresses in depression. *European Psychiatry*.

[64] Jiménez-Fernández S., Gurpegui M., Díaz-Atienza F., Pérez-Costillas L., Gerstenberg M., Correll C. U. (2015). Oxidative stress and antioxidant parameters in patients with major depressive disorder compared to healthy controls before and after antidepressant Treatment. *Journal of Clinical Psychiatry*.

[65] Narayanan S. V., Dave K. R., Perez-Pinzon M. A. (2018). Ischemic preconditioning protects astrocytes against oxygen glucose deprivation via the nuclear erythroid 2-related factor 2 pathway. *Translational Stroke Research*.

[66] Yao Y., Miao W., Liu Z. (2016). Dimethyl fumarate and monomethyl fumarate promote post-ischemic recovery in mice. *Translational Stroke Research*.

[67] Lien L. M., Chiou H. Y., Yeh H. L. (2017). Significant association between low mitochondrial DNA content in peripheral blood leukocytes and ischemic stroke. *Journal of the American Heart Association*.

[68] Wang A., Cui Y., Meng X. (2018). The relationship between oxidized low-density lipoprotein and the NIHSS score among patients with acute ischemic stroke: the SOS-stroke study. *Atherosclerosis*.

[69] Wang A., Liu J., Meng X. (2018). Association between oxidized low‐density lipoprotein and cognitive impairment in patients with ischemic stroke. *European Journal of Neurology*.

[70] Anwar M. A., Eid A. H. (2016). Determination of vascular reactivity of middle cerebral arteries from stroke and spinal cord Injury animal models using pressure myography. *Methods in Molecular Biology*.

[71] Xing C., Hayakawa K., Lo E. H. (2017). Mechanisms, imaging, and therapy in stroke recovery. *Translational Stroke Research*.

[72] Yang B., Xu B., Zhao H. (2018). Dioscin protects against coronary heart disease by reducing oxidative stress and inflammation via Sirt1/Nrf2 and p38 MAPK pathways. *Molecular Medicine Reports*.

[73] Zhang Z., Jiang F., Zeng L., Wang X., Tu S. (2018). PHACTR1 regulates oxidative stress and inflammation to coronary artery endothelial cells via interaction with NF-*κ*B/p65. *Atherosclerosis*.

[74] Zhang J., Wang M., Li Z. (2016). NADPH oxidase activation played a critical role in the oxidative stress process in stable coronary artery disease. *American Journal of Translational Research*.

[75] Ibi M., Liu J., Arakawa N. (2017). Depressive-like behaviors are regulated by NOX1/NADPH oxidase by redox modification of NMDA receptor 1. *The Journal of Neuroscience*.

[76] Pasquali M. A., Harlow B. L., Soares C. N. (2018). A longitudinal study of neurotrophic, oxidative, and inflammatory markers in first-onset depression in midlife women. *European Archives of Psychiatry and Clinical Neuroscience*.

[77] Liu Z., Zhu Z., Zhao J. (2017). Malondialdehyde: a novel predictive biomarker for post-stroke depression. *Journal of Affective Disorders*.

[78] Nabavi S., Habtemariam S., di Lorenzo A. (2016). Post-stroke depression modulation and in vivo antioxidant activity of gallic acid and its synthetic derivatives in a murine model system. *Nutrients*.

[79] Nabavi S. M., Nabavi S. F., Sureda A. (2018). The water extract of tutsan (*Hypericum androsaemum* L.) red berries exerts antidepressive-like effects and *in vivo* antioxidant activity in a mouse model of post-stroke depression. *Biomedicine & Pharmacotherapy*.

[80] Di Lorenzo A., Nabavi S. F., Sureda A. (2016). Antidepressive-like effects and antioxidant activity of green tea and GABA green tea in a mouse model of post-stroke depression. *Molecular Nutrition & Food Research*.

[81] Mazereeuw G., Herrmann N., Andreazza A. C. (2017). Oxidative stress predicts depressive symptom changes with omega-3 fatty acid treatment in coronary artery disease patients. *Brain, Behavior, and Immunity*.

[82] Stafford L., Berk M. (2011). The use of statins after a cardiac intervention is associated with reduced risk of subsequent Depression. *Journal of Clinical Psychiatry*.

[83] Ahn J., Shin M., Kim D. (2019). Antioxidant properties of fucoidan alleviate acceleration and exacerbation of hippocampal neuronal death following transient global cerebral ischemia in high-fat diet-induced obese gerbils. *International Journal of Molecular Sciences*.

[84] Gramlich Y., Daiber A., Buschmann K. (2018). Oxidative stress in cardiac tissue of patients undergoing coronary artery bypass graft surgery: the effects of overweight and obesity. *Oxidative Medicine and Cellular Longevity*.

[85] Kurhe Y., Mahesh R. (2015). Ondansetron attenuates co-morbid depression and anxiety associated with obesity by inhibiting the biochemical alterations and improving serotonergic neurotransmission. *Pharmacology, Biochemistry, and Behavior*.

[86] Gao W., Wang W., Zhang J. (2019). Allicin ameliorates obesity comorbid depressive-like behaviors: involvement of the oxidative stress, mitochondrial function, autophagy, insulin resistance and NOX/Nrf2 imbalance in mice. *Metabolic Brain Disease*.

[87] Catalano P. M., Shankar K. (2017). Obesity and pregnancy: mechanisms of short term and long term adverse consequences for mother and child. *BMJ*.

[88] Roomruangwong C., Anderson G., Berk M., Stoyanov D., Carvalho A. F., Maes M. (2018). A neuro-immune, neuro-oxidative and neuro-nitrosative model of prenatal and postpartum depression. *Progress in Neuro-Psychopharmacology & Biological Psychiatry*.

[89] Li P. C., Liu L. F., Jou M. J., Wang H. K. (2016). The GLP-1 receptor agonists exendin-4 and liraglutide alleviate oxidative stress and cognitive and micturition deficits induced by middle cerebral artery occlusion in diabetic mice. *BMC Neuroscience*.

[90] Zhao M., Li X. W., Chen D. Z. (2019). Neuro-protective role of metformin in patients with acute stroke and type 2 diabetes mellitus via AMPK/mammalian target of rapamycin (mTOR) signaling pathway and oxidative stress. *Medical Science Monitor*.

[91] Thomas M. C., Woodward M., Li Q. (2018). Relationship between plasma 8-OH-deoxyguanosine and cardiovascular disease and survival in type 2 diabetes mellitus: results from the ADVANCE trial. *Journal of the American Heart Association*.

[92] Rebai R., Jasmin L., Boudah A. (2017). The antidepressant effect of melatonin and fluoxetine in diabetic rats is associated with a reduction of the oxidative stress in the prefrontal and hippocampal cortices. *Brain Research Bulletin*.

[93] Duicu O. M., Lighezan R., Sturza A. (2016). Assessment of mitochondrial dysfunction and monoamine oxidase contribution to oxidative stress in human diabetic hearts. *Oxidative Medicine and Cellular Longevity*.

[94] Ngarashi D., Fujikawa K., Ferdaus M. Z., Zahid H. M., Ohara H., Nabika T. (2019). Dual inhibition of NADPH oxidases and xanthine oxidase potently prevents salt-induced stroke in stroke-prone spontaneously hypertensive rats. *Hypertension Research*.

[95] Lan C., Chen X., Zhang Y. (2018). Curcumin prevents strokes in stroke-prone spontaneously hypertensive rats by improving vascular endothelial function. *BMC Cardiovascular Disorders*.

[96] Rubattu S., Cotugno M., Bianchi F. (2017). A differential expression of uncoupling protein-2 associates with renal damage in stroke-resistant spontaneously hypertensive rat/stroke-prone spontaneously hypertensive rat-derived stroke congenic lines. *Journal of Hypertension*.

[97] Dos Santos R. L., Dellacqua L. O., Delgado N. T. (2016). Pomegranate peel extract attenuates oxidative stress by decreasing coronary angiotensin-converting enzyme (ACE) activity in hypertensive female rats. *Journal of Toxicology and Environmental Health. Part A*.

[98] Koibuchi N., Hasegawa Y., Katayama T. (2014). DPP-4 inhibitor linagliptin ameliorates cardiovascular injury in salt-sensitive hypertensive rats independently of blood glucose and blood pressure. *Cardiovascular Diabetology*.

[99] Robaczewska J., Kędziora-Kornatowska K., Kucharski R. (2016). Decreased expression of heme oxygenase is associated with depressive symptoms and may contribute to depressive and hypertensive comorbidity. *Redox Report*.

[100] Zhang F., Yan C., Wei C. (2018). Vinpocetine inhibits NF-*κ*B-Dependent inflammation in acute ischemic stroke patients. *Translational Stroke Research*.

[101] Wirtz P. H., von Kanel R. (2017). Psychological stress, inflammation, and coronary heart disease. *Current Cardiology Reports*.

[102] Shao A., Zhu Z., Li L., Zhang S., Zhang J. (2019). Emerging therapeutic targets associated with the immune system in patients with intracerebral haemorrhage (ICH): from mechanisms to translation. *eBioMedicine*.

[103] Ferrarese C., Mascarucci P., Zoia C. (1999). Increased cytokine release from peripheral blood cells after acute stroke. *Journal of Cerebral Blood Flow and Metabolism*.

[104] Lalancette-Hebert M., Gowing G., Simard A., Weng Y. C., Kriz J. (2007). Selective ablation of proliferating microglial cells exacerbates ischemic injury in the brain. *The Journal of Neuroscience*.

[105] Hurn P. D., Subramanian S., Parker S. M. (2007). T- and B-cell-deficient mice with experimental stroke have reduced lesion size and inflammation. *Journal of Cerebral Blood Flow and Metabolism*.

[106] Teague H., Mehta N. N. (2016). The link between inflammatory disorders and coronary heart disease: a look at recent studies and novel drugs in development. *Current Atherosclerosis Reports*.

[107] Kim S. W., Kang H. J., Bae K. Y. (2018). Interactions between pro-inflammatory cytokines and statins on depression in patients with acute coronary syndrome. *Progress in Neuro-Psychopharmacology & Biological Psychiatry*.

[108] Rozing M. P., Veerhuis R., Westendorp R. G. J. (2019). Inflammation in older subjects with early- and late-onset depression in the NESDO study: a cross-sectional and longitudinal case-only design. *Psychoneuroendocrinology*.

[109] Alcocer-Gómez E., de Miguel M., Casas-Barquero N. (2014). NLRP3 inflammasome is activated in mononuclear blood cells from patients with major depressive disorder. *Brain Behavior and Immunity*.

[110] Leday G. G. R., Vértes P. E., Richardson S. (2018). Replicable and coupled changes in innate and adaptive immune gene expression in two case-control studies of blood microarrays in major depressive disorder. *Biological Psychiatry*.

[111] Jiao J. T., Cheng C., Ma Y. J. (2016). Association between inflammatory cytokines and the risk of post-stroke depression, and the effect of depression on outcomes of patients with ischemic stroke in a 2-year prospective study. *Experimental and Therapeutic Medicine*.

[112] Nikkheslat N., Zunszain P. A., Horowitz M. A. (2015). Insufficient glucocorticoid signaling and elevated inflammation in coronary heart disease patients with comorbid depression. *Brain, Behavior, and Immunity*.

[113] Gilbert K., Bernier J., Godbout R., Rousseau G. (2014). Resolvin D1, a metabolite of omega-3 polyunsaturated fatty acid, decreases post-myocardial infarct depression. *Marine Drugs*.

[114] Götschel F., Kern C., Lang S. (2008). Inhibition of GSK3 differentially modulates NF-*κ*B, CREB, AP-1 and *β*-catenin signaling in hepatocytes, but fails to promote TNF-*α*-induced apoptosis. *Experimental Cell Research*.

[115] Rana A. K., Singh D. (2018). Targeting glycogen synthase kinase-3 for oxidative stress and neuroinflammation: opportunities, challenges and future directions for cerebral stroke management. *Neuropharmacology*.

[116] Zhang Q., Raoof M., Chen Y. (2010). Circulating mitochondrial DAMPs cause inflammatory responses to injury. *Nature*.

[117] Hornung V., Bauernfeind F., Halle A. (2008). Silica crystals and aluminum salts activate the NALP3 inflammasome through phagosomal destabilization. *Nature Immunology*.

[118] Ni Y., Gu W. W., Liu Z. H. (2018). RIP1K contributes to neuronal and astrocytic cell death in ischemic stroke via activating autophagic-lysosomal pathway. *Neuroscience*.

[119] Balaganapathy P., Baik S. H., Mallilankaraman K., Sobey C. G., Jo D. G., Arumugam T. V. (2018). Interplay between notch and p53 promotes neuronal cell death in ischemic stroke. *Journal of Cerebral Blood Flow and Metabolism*.

[120] Chen B., Wang G., Li W. (2017). Memantine attenuates cell apoptosis by suppressing the calpain-caspase-3 pathway in an experimental model of ischemic stroke. *Experimental Cell Research*.

[121] Zhu Y., Yang T., Duan J., Mu N., Zhang T. (2019). MALAT1/miR-15b-5p/MAPK1 mediates endothelial progenitor cells autophagy and affects coronary atherosclerotic heart disease via mTOR signaling pathway. *Aging (Albany NY)*.

[122] Wu P., Yuan X., Li F. (2017). Myocardial upregulation of cathepsin D by ischemic heart disease promotes autophagic flux and protects against cardiac remodeling and heart failure. *Circulation. Heart Failure*.

[123] Su H. H., Chu Y. C., Liao J. M. (2017). Phellinus linteus Mycelium alleviates myocardial ischemia-reperfusion injury through autophagic regulation. *Frontiers in Pharmacology*.

[124] Wen J., Chen C. H., Stock A., Doerner J., Gulinello M., Putterman C. (2016). Intracerebroventricular administration of TNF-like weak inducer of apoptosis induces depression-like behavior and cognitive dysfunction in non-autoimmune mice. *Brain, Behavior, and Immunity*.

[125] Xiao X., Shang X., Zhai B., Zhang H., Zhang T. (2018). Nicotine alleviates chronic stress-induced anxiety and depressive-like behavior and hippocampal neuropathology via regulating autophagy signaling. *Neurochemistry International*.

[126] Liu S., Han S., Dai Q., Li S., Li J. (2018). BICAO-induced ischaemia caused depressive-like behaviours and caspase-8/-9-dependent brain regional neural cell apoptosis in mice. *Stroke Vasc Neurol*.

[127] Wang Y., Zhang H., Chai F., Liu X., Berk M. (2014). The effects of escitalopram on myocardial apoptosis and the expression of Bax and Bcl-2 during myocardial ischemia/reperfusion in a model of rats with depression. *BMC Psychiatry*.

[128] Vandenabeele P., Galluzzi L., vanden Berghe T., Kroemer G. (2010). Molecular mechanisms of necroptosis: an ordered cellular explosion. *Nature Reviews Molecular Cell Biology*.

[129] Culmsee C., Zhu C., Landshamer S. (2005). Apoptosis-inducing factor triggered by poly(ADP-ribose) polymerase and Bid mediates neuronal cell death after oxygen-glucose deprivation and focal cerebral ischemia. *The Journal of Neuroscience*.

[130] McKernan D. P., Dinan T. G., Cryan J. F. (2009). "Killing the Blues": A role for cellular suicide (apoptosis) in depression and the antidepressant response?. *Progress in Neurobiology*.

[131] Wang P., Shao B. Z., Deng Z., Chen S., Yue Z., Miao C. Y. (2018). Autophagy in ischemic stroke. *Progress in Neurobiology*.

[132] Benakis C., Brea D., Caballero S. (2016). Commensal microbiota affects ischemic stroke outcome by regulating intestinal *γδ* T cells. *Nature Medicine*.

[133] Dou Z., Rong X., Zhao E., Zhang L., Lv Y. (2019). Neuroprotection of resveratrol against focal cerebral ischemia/reperfusion injury in mice through a mechanism targeting gut-brain axis. *Cellular and Molecular Neurobiology*.

[134] Lu X., Wang Y., Liu C., Wang Y. (2017). Depressive disorder and gastrointestinal dysfunction after myocardial infarct are associated with abnormal tryptophan-5-hydroxytryptamine metabolism in rats. *PLoS One*.

[135] Valles-Colomer M., Falony G., Darzi Y. (2019). The neuroactive potential of the human gut microbiota in quality of life and depression. *Nature Microbiology*.

[136] Bailey M. T., Cryan J. F. (2017). The microbiome as a key regulator of brain, behavior and immunity: commentary on the 2017 named series. *Brain, Behavior, and Immunity*.

[137] Rao M., Gershon M. D. (2016). The bowel and beyond: the enteric nervous system in neurological disorders. *Nature Reviews Gastroenterology & Hepatology*.

[138] Dumitrescu L., Popescu-Olaru I., Cozma L. (2018). Oxidative stress and the microbiota-gut-brain axis. *Oxidative Medicine and Cellular Longevity*.

[139] Wen S. W., Wong C. H. Y. (2017). An unexplored brain-gut microbiota axis in stroke. *Gut Microbes*.

[140] Slyepchenko A., Maes M., Jacka F. N. (2017). Gut microbiota, bacterial translocation, and interactions with diet: pathophysiological links between major depressive disorder and non-communicable medical comorbidities. *Psychotherapy and Psychosomatics*.

[141] Gomes C., Martinho F. C., Barbosa D. S. (2018). Increased root canal endotoxin levels are associated with chronic apical periodontitis, increased oxidative and nitrosative stress, major depression, severity of depression, and a lowered quality of life. *Molecular Neurobiology*.

